# Perceived stress and high fat intake: A study in a sample of undergraduate students

**DOI:** 10.1371/journal.pone.0192827

**Published:** 2018-03-09

**Authors:** E. Jair Vidal, Daily Alvarez, Dalia Martinez-Velarde, Lorena Vidal-Damas, Kelly A. Yuncar-Rojas, Alesia Julca-Malca, Antonio Bernabe-Ortiz

**Affiliations:** 1 School of Medicine, Universidad Peruana de Ciencias Aplicadas, Lima, Perú; 2 CRONICAS Center of Excellence in Chronic Diseases, Universidad Peruana Cayetano Heredia, Lima, Peru; 3 Faculty of Epidemiology and Population Health, London School of Hygiene and Tropical Medicine, London, United Kingdom; Fondazione Toscana Gabriele Monasterio, ITALY

## Abstract

**Objectives:**

Different studies have reported the association between perceived stress and unhealthy diet choices. We aimed to determine whether there is a relationship between perceived stress and fat intake among undergraduate medical students.

**Methods/Principal findings:**

A cross-sectional study was performed including first-year medical students. The outcome of interest was the self-report of fat intake assessed using the Block Screening Questionnaire for Fat Intake (high vs. low intake), whereas the exposure was perceived stress (low/normal vs. high levels). The prevalence of high fat intake was estimated and the association of interest was determined using prevalence ratios (PR) and 95% confidence intervals (95%CI). Models were created utilizing Poisson regression with robust standard errors.

Data from 523 students were analyzed, 52.0% female, mean age 19.0 (SD 1.7) years. The prevalence of high fat intake was 42.4% (CI: 38.2%–46.7%). In multivariate model and compared with those with lowest levels of stress, those in the middle (PR = 1.59; 95%CI: 1.20–2.12) and highest (PR = 1.92; 95%CI: 1.46–2.53) categories of perceived stress had greater prevalence of fat intake. Gender was an effect modifier of this association (p = 0.008).

**Conclusions:**

Greater levels of perceived stress were associated with higher fat intake, and this association was stronger among males. More than 40% of students reported having high fat consumption. Our results suggest the need to implement strategies that promote decreased fat intake.

## Introduction

Worldwide, there is a rising burden of mental health disorders [1]. Stress, defined as “a complex physiological state that embodies a range of integrative physiological and behavioral processes that occur when there is a real or perceived threat to homeostasis” [2], can be related to the pressures of work, family and other daily responsibilities, which might affect many aspects of life (i.e. physical, behavioral and psychiatric manifestations) explained by sustained, excessive secretion and effect of major mediators of stress. Entering college or university can be an exciting, yet a stressful event for many young individuals as they face trying to adapt to changes in academic workloads. This is more patent in medical students. For example, a previous systematic review reported high levels of psychological distress among medical students compared to the general population and age-matched peers by years of training [3]. Moreover, a number of factors, including academic pressure, workload, sleep deprivation, among others, were considered as the main causes of higher level of stress in this population [4, 5] as well as changes in nutrition patterns.

Diet and nutrition are important factors in the promotion and maintenance of good health throughout the entire life course [6]. Poor nutrition can lead to chronic non communicable conditions, and unhealthy lifestyles (i.e. poor diet patterns, lack of physical activity, etc.) have been known for many years to play a key role as a risk factor for chronic diseases. Thus, it has been demonstrated that the adoption of fat-rich diets (especially those related to those linked to high energy), as well as the low levels of physical activity, increases the risk of obesity, and furthermore, the developing of chronic conditions, including diabetes, hypertension, among others [6].

Recent studies have reported that stress increase levels of cortisol among individuals, inducing to an increment in the intake of high-fat foods to control this stress [7–9]. Moreover, a study using animal models found rewarding properties of sweet palatable foods on stress relief, in addition that long-term intake of dietary fat, and their subsequent removal, can promote stress-related outcomes [10]. A previous study enrolling 40 women reported that high-stressed females preferred sweet, high-fat food more than did low-stressed women [11]. As levels of stress are different according to gender [12], stress might have a differential impact on the decision of eating unhealthy food and this association might vary when being assessed by gender.

Some papers have shown the association between perceived stress and unhealthy diet choices among students. For example, in one of the manuscripts, snacking behavior was increased by stress in 73% of respondents, regardless gender or dieting status [13], whereas *Papier et al* demonstrated a difference in food selection between stressed male and female students, with stress being a more significant predictor of unhealthy food selection among male students [14]. However, limited information is available from developing countries such as Peru, a country under current nutritional transition [15]. Moreover, obesity rates have markedly increased the last 20 years, especially among the young adults [16–18]. Therefore, this study aimed to determine whether there is a relationship between perceived stress levels and fat intake among undergraduate medical students, and to verify if this relationship depends on gender. In addition, the prevalence of high intake of fat was also estimated in this population.

## Materials and methods

### Study design and participants

A cross-sectional study was conducted enrolling a random sample of undergraduate students at the Universidad Peruana de Ciencias Aplicadas (UPC), in Lima, Peru. Potential participants were those registered in the first three years of the School of Medicine during the first semester of 2015.

### Variables definition

The outcome of interest was the self-reported amount of fat intake, defined according to the Block Screening Questionnaire for Fat Intake [19]. This brief food frequency questionnaire, developed by Block et al from analyses of NHANES II, was designed for screening purposes. The food items included in this screening tool are a subset of those found in the 100-item Health Habits and History Questionnaire [20]. The tool consists of 15 questions regarding the eating habits over the past year and lists foods with a high fat content. Each question has five response options (less than once per month, 2–3 times per month, 1–2 times per week, 3–4 times per week, and ≥5 times per week) scored from 0 to 4 points, respectively. The Spanish version of this tool, validated and adapted by the Institute of Nutrition of Central America and Panama (INCAP), was used in this study [21]. Overall score is calculated by adding individual responses and then split into two categories: low fat intake (<25 points) and high fat intake (≥25 points). Additionally, those participants with ≥27 points are considered to consume a diet with very high fat content.

The exposure of interest was perceived stress, assessed using the Cohen’s Perceived Stress Scale, previously validated in Spanish [22]. A total of 14 items assesses the perception of stress during the last month, with five options of response for each question: never, almost never, sometimes, fairly often, and very often. Global score ranges from 0 to 56, with a higher score indicating higher levels of perceived stress [23]. As a usual cutoff has not been reported, the variable was categorized in tertiles and then grouped in low/normal vs. high levels of stress for analysis.

Other variables included in the analysis were gender, age (<18 and ≥18 years), place of birth (Lima/Callao vs. migrant), study year (first, second and third), living alone (no/yes), and the presence of depressive symptoms, assessed by using the first two screening questions of the Patient Health Questionnaire (PHQ-9) [24].

### Study procedures and data collection

Undergraduate students were contacted in their classroom before lectures. Informed consent was administered before starting data collection activities. A self-applied questionnaire was provided to students including information regarding socio-demographic data, the Block Screening Questionnaire for Fat Intake and the Cohen’s Perceived Stress Scale. Responding to the questionnaire took on average 15 minutes, and after that process, a carefully review of the survey was carried out with the participant to guarantee appropriate completeness.

### Sample size

Sample size was calculated using Power and Sample Size (PASS 2008, NCSS, Utah, US). Assuming a power of 80% and a level of significance of 5%, 514 participants were required to detect a difference in the prevalence of high fat intake among those with higher levels of stress and low levels of stress of 15% (i.e. 35% vs. 20%, respectively).

### Statistical analysis

After data collection, a double data entry process was performed using Microsoft Excel for Windows. Then, data was transferred to STATA 13 (STATA Corp, College Station, TX, US) for statistical analysis. First, a description of the study population was performed using proportions to compare characteristics according to the exposure and outcome. Chi-squared test was used to compare categorical variables. Then, prevalence and 95% confidence intervals (95%CI) of high fat and very high fat intake were estimated. Prevalence ratios (PR) and 95%CI were calculated using Poisson regression models with robust standard errors [25, 26]. Gender was assessed as a potential effect modifier of the proposed association. Models were fitted including potential confounders.

### Ethics

The study and procedures were approved by the Ethical Committee of the Universidad Peruana de Ciencias Aplicadas (UPC), Lima, Peru. As a group of participants were under 18 years, an overall verbal consent was requested to the School of Medicine and courses coordinators before survey application. Written informed consent was waived as information to be collected was not sensitive and, as a result, only oral consent was obtained directly from participants. Fieldworkers signed in the informed consent format to document participant consent. Data was collected without personal identifiers to guarantee confidentiality.

## Results

### Characteristics of the study population

From a total of 1178 medical students registered, 528 individuals were randomly invited to participate in the study, but 5 (0.9%) refuse to participate; thus, data from 523 students were analyzed, 272 (52.0%) female, mean age 19.0 (SD: 1.8) years.

### Perceived stress

The overall mean level of perceived stress was 25.9 (SD: 6.5). When split according to stress levels, the mean of perceived stress was 18.8 (SD: 5.1, range: 3–24 points) in the low group, 26.7 (SD: 1.1, range: 25–28 points) in the middle group, and 32.3 (SD: 3.2, range: 29–45 points) in the high stress group. Characteristics of the study population according to perceived stress tertiles is shown in Table 1. Of note, perceived stress was associated with gender (p = 0.03) and study year (p<0.001).

**Table 1:** Characteristics of the study population according to perceived stress levels.

	Perceived stress			p-value*
	Low tertile	Middle tertile	High tertile	
	(n = 175)	(n = 177)	(n = 171)	
***Gender***				
Female	83 (47.4%)	86 (48.6%)	103 (60.2%)	0.03
Male	92 (52.6%)	91 (51.4%)	68 (39.8%)	
***Age***				
≥ 18 years	160 (91.4%)	151 (85.3%)	150 (87.7%)	0.20
< 18 years	15 (8.6%)	26 (14.7%)	21 (12.3%)	
**Place of birth**				
Lima/Callao	122 (69.7%)	129 (72.9%)	123 (71.9%)	0.80
Migrant	53 (30.3%)	48 (27.1%)	48 (28.1%)	
**Study year**				
First	50 (28.6%)	86 (48.6%)	70 (40.9%)	<0.001
Second	72 (41.1%)	68 (38.4%)	75 (43.9%)	
Third	53 (30.3%)	23 (13.0%)	26 (15.2%)	
**Live alone**				
No	142 (81.1%)	136 (76.8%)	138 (80.7%)	0.55
Yes	33 (18.9%)	41 (23.2%)	33 (19.3%)	
**Depressive symptoms**				
No	172 (98.3%)	167 (94.4%)	160 (93.6%)	0.08
Yes	3 (1.7%)	10 (5.6%)	11 (6.4%)	

Results may not add due to missing values

*Chi-squared test was used to calculate p-values

### Prevalence of fat intake

The prevalence of high fat intake was 42.4% (95%CI: 38.2%–46.7%) and about 30.4% (95%CI: 26.5%–34.5%) reported having a very high fat intake. In bivariable model (Table 2), high fat intake was more frequent among males (47.4%) than females (37.9%, p = 0.03), and greater among younger (62.9%) than older individuals (37.9%, p = 0.001). The proportion of subjects reporting high intake of fat was greater among those in the highest tertile of perceived stress (53.2%) compared to those in the middle tertile (46.3%) and the lowest tertile (28.0%, p-value<0.001).

**Table 2:** Characteristics of the study population according to self-reported fat intake.

	Self-reported fat intake		
	Low intake	High intake	p-value*
	(n = 301)	(n = 222)	
***Gender***			
Female	169 (62.1%)	103 (37.9%)	0.03
Male	132 (52.6%)	119 (47.4%)	
***Age***			
≥ 18 years	278 (60.3%)	183 (39.7%)	0.001
< 18 years	23 (37.1%)	39 (62.9%)	
**Place of birth**			
Lima/Callao	213 (57.0%)	161 (43.1%)	0.66
Migrant	88 (59.1%)	61 (40.9%)	
**Study year**			
First	61 (29.6%)	145 (70.4%)	<0.001
Second	159 (74.0%)	56 (26.1%)	
Third	81 (79.4%)	21 (20.6%)	
**Live alone**			
No	242 (58.2%)	174 (41.8%)	0.57
Yes	59 (55.1%)	48 (44.9%)	
**Depressive symptoms**			
No	288 (57.7%)	211 (42.3%)	0.73
Yes	13 (54.2%)	11 (45.8%)	
**Perceived stress**			
Low	126 (72.0%)	49 (28.0%)	<0.001
Middle	95 (53.7%)	82 (46.3%)	
High	80 (46.8%)	91 (53.2%)	

Percentages are calculated in rows. Results may not add due to missing values

*Chi-squared test was used to calculate p-values

### Association between perceived stress and fat intake

The correlation between fat intake and perceived stress as numerical variables was significant (ρ = 0.15, p-value = 0.005), and there was evidence of an increasing trend of fat intake associated with greater perceived stress (p<0.001). Thus, when compared to the lowest level, those in the middle (PR = 1.59; 95%CI: 1.20–2.12) and highest (PR = 1.92; 95%CI: 1.46–2.53) categories of perceived stress had greater prevalence of fat intake after controlling for gender, age, place of birth, living alone, and depressive symptoms (Table 3).

**Table 3:** Overall association between fat intake and perceived stress: Crude and adjusted models.

	Crude model	Adjusted model*
	PR (95%CI)	PR (95%CI)
***Gender***		
Female	1 (Reference)	1 (Reference)
Male	**1.25 (1.02–1.53)**	**1.33 (1.09–2.53)**
***Age***		
≥ 18 years	1 (Reference)	1 (Reference)
< 18 years	**1.58 (1.27–1.98)**	**1.55 (1.24–1.93)**
**Place of birth**		
Lima/Callao	1 (Reference)	1 (Reference)
Migrant	0.95 (0.76–1.19)	0.93 (0.75–1.16)
**Live alone**		
No	1 (Reference)	1 (Reference)
Yes	1.07 (0.84–1.36)	1.06 (0.84–1.33)
**Depressive symptoms**		
No	1 (Reference)	1 (Reference)
Yes	1.08 (0.69–1.70)	1.05 (0.67–1.64)
**Perceived stress**		
Low	1 (Reference)	1 (Reference)
Middle	**1.65 (1.24–2.20)**	**1.59 (1.20–2.12)**
High	**1.90 (1.44–2.51)**	**1.92 (1.46–2.53)**

Bold estimates are significant (p<0.05)

* The model is adjusted for all the variables listed in the table.

Finally, gender was an effect modifier of this association (p = 0.008): the association of interest was stronger among males compared to females (See Table 4).

**Table 4:** Association between fat intake and perceived stress by gender: Crude and adjusted models.

	Sex: Females		Sex: Males	
	Crude model	Adjusted model*	Crude model	Adjusted model*
	PR (95%CI)	PR (95%CI)	PR (95%CI)	PR (95%CI)
***Age***				
≥ 18 years	1 (Reference)	1 (Reference)	1 (Reference)	1 (Reference)
< 18 years	**1.88 (1.39–2.55)**	**1.88 (1.39–2.55)**	1.34 (0.96–1.88)	1.19 (0.85–1.66)
**Place of birth**				
Lima/Callao	1 (Reference)	1 (Reference)	1 (Reference)	1 (Reference)
Migrant	0.94 (0.66–1.34)	0.95 (0.66–1.35)	0.94 (0.70–1.26)	0.96 (0.72–1.27)
**Live alone**				
No	1 (Reference)	1 (Reference)	1 (Reference)	1 (Reference)
Yes	1.02 (0.69–1.50)	0.99 (0.67–1.45)	1.10 (0.81–1.48)	1.04 (0.78–1.38)
**Depressive symptoms**				
No	1 (Reference)	1 (Reference)	1 (Reference)	1 (Reference)
Yes	1.44 (0.89–2.31)	1.35 (0.85–2.14)	0.60 (0.18–1.94)	0.55 (0.18–1.70)
**Perceived stress**				
Low	1 (Reference)	1 (Reference)	1 (Reference)	1 (Reference)
Middle	1.47 (0.95–2.26)	1.40 (0.92–2.14)	**1.83 (1.25–2.68)**	**1.82 (1.24–2.67)**
High	**1.58 (1.04–2.38)**	**1.55 (1.03–2.32)**	**2.39 (1.66–3.45)**	**2.37 (1.64–3.42)**

Bold estimates are significant (p<0.05)

* The model is adjusted for all the variables listed in the table.

## Discussion

### Main findings

Based on our results, perceived stress was positively associated with an increased intake of fat-rich foods after controlling for gender, age, place of birth, living alone, and depressive symptoms. Moreover, gender was an effect modifier of this association: males had greater probability of consuming high amount of fat when compared to females. Additionally, more than 40% of undergraduate students reported having a high intake of fat.

### Comparison with previous studies

Our results are consistent with two previous longitudinal studies [27, 28]. One of them, a secondary analysis of an intervention, reported that greater perceived stress was associated with lower levels of eating awareness and physical activity. Moreover, among those who had low levels of eating awareness, higher levels of perceived stress were associated with fewer servings of fruit and vegetables and greater consumption of fast food meals [27]. In the other longitudinal study, conducted only among women, greater levels of perceived stress was associated with an increased body mass index. Besides, longitudinal associations were found between stress and lower leisure-time physical activity, and more frequent fast food consumption and increased television viewing time [28].

Similarly, our results are consistent with some aforementioned cross-sectional studies [13, 14]. A previous manuscript reported that low levels of perceived stress and high diet self-efficacy were associated with lowest levels of fat and sodium intake among students [29]. Interestingly, these findings were driven mainly by females. Moreover, a study including first-year British students tested the hypothesis that stress plays a role on weight gain. Students reported a significant weight increase (1.5 kg on average) but with a great variation in results: 55% reported weight gain, 12% weight loss, and 33% remaining stable. Apparently, findings were due to reduction of exercise, although students reporting more snacking at university had greater reported weight gain [8]. On the other hand, in a study carried out among first-year students from three European countries, the perceived stress was greater in those who reported higher consumption of sweets and fast foods; however, this association was present among females but not males [7]. A potential explanation for these discrepancies may include female perceptions of self- and body-image and self-esteem, which may be associated with food consumption [30], and can be higher in our young population. Our results then suggest that other mechanisms might be present in the decision of consuming high-fat food. Thus, future interventions need to focus in the gender of potential participants. Finally, the reverse effect has been also reported: some kind of foods can increase the probability of having stress: ready-to-eat and snack food showed positively significant correlation with perceived stress in humans [12] as well as animals [10].

As previously reported, stress apparently can lead to changes in food choices; thus, individuals prefers especially meals that are high in fat and less healthy [9]. Loss of control mechanisms (i.e. endogenous opioid release secondary to palatable food) to avoid this kind of food has been proposed, but the increased levels of cortisol secondary to stress might promote individuals to increase the consumption of unhealthy food [11, 31]. A previous study reported that increased cortisol secretion due to stress, reduced dietary restraint and increased caloric intake [32], accounting for 73% of the variance in change in body mass index. As a result, other studies are needed to better understand the link between stress and food decisions, and hence overweight and obesity.

Our findings also showed that more than 40% of medical students reported having a high-fat intake as previously reported in other settings [33, 34]. One of these reports found that 45% of students consumed fast food twice a week and 35% used the frying oils for cooking most of the times [33], whereas in other study, around 75% of students reported daily intake of high fat diet [35]. When compared to general population, young people, comparable in age to those included in this study, reported a intake of fat of 34.3% of the food consumed [36]. Thus, apparently medical students tend to eat more fat-enriched food than those from general population. As a great proportion of students reported consuming high levels of fat-enriched food, interventions are also needed. Potential strategies might include for example, increasing students’ awareness about the potential long-term consequences of high-fat diets. Health information and communication strategies about the benefits of healthy eating and physical activity can be useful [37] as well as the potential use of technology (SMS through smartphones or mHealth), especially for young individuals. On the other hand, the reduction of the offer of fat-rich food to students as well as the increment of prices of unhealthy foods, especially in the university where students stay for several hours has been shown to be cost effective and reducing unhealthy foods as part of rewards or celebrations in the university. For example, Caparosa et al found substantial amounts of unhealthy foods as part of rewards, celebrations or other activities [38], leveraging the need of school nutrition policies as a potential intervention. Nevertheless, future studies are needed to assess the potential impact of these strategies in students’ health.

### Strengths and limitations

This study demonstrates a relationship between high levels of perceived stress and greater fat intake using a sample of undergraduate medical students. However, this study has several limitations. First, due to the cross-sectional nature, only association and not causality can be determined. In addition, the sample included in the study might not be representative of the total medical population in our country. Our findings were only estimated using a sample of students from a private university and therefore, results may not be extrapolated to public institutions. However, other studies have shown the same association and our results are similar to them [13, 27–29]. Second, fat intake was determined by self-report; as a result, social desirability bias might arise especially in the more advanced students. Nevertheless, self-reported rates of fat intake are high in this sample of students. Third, some reverse causality might appear as there is a temporal mismatch between information from the Block Screening Questionnaire for Fat Intake (information based on last year) and the Cohen’s Perceived Stress of Scale (information based on last four weeks). However, previous studies have shown the direction of the association as we have proposed in this study [27, 28]. Fourth, although we adjusted for the presence of depressive symptoms in our multivariable model, only the first two questions of the PHQ-9 were used. As a result, there is the possibility that misclassification (i.e. some individuals with depressive symptoms were not detected by the screening tool) could affect our results. Fifth, body mass index (or any other obesity-related markers) was not collected during the study; thus, adjustment for anthropometrical marker was not possible. Finally, other variables, potential confounders, such as socioeconomic status, parent’s education level, other foods intake, and lipid profile (cholesterol and triglycerides), were not assessed. In addition, physical activity and walking, which have been associated with perceived stress [27], were not evaluated in this study. Furthermore, other studies, prospective in nature, are needed to corroborate our findings and their potential impact on students’ health.

### Conclusions

Greater levels of perceived stress were associated with higher fat intake, and this association was stronger among males than females. More than 40% of students reported having high fat consumption. There is a need to implement appropriate strategies to inform students regarding the benefits of healthy food and the negative effects of the fat-rich foods.
